# Healthcare workers’ industrial action in Nigeria: a cross-sectional survey of Nigerian physicians

**DOI:** 10.1186/s12960-018-0322-8

**Published:** 2018-10-17

**Authors:** Obinna Ositadimma Oleribe, Deborah Udofia, Olabisi Oladipo, Temitope Arike Ishola, Simon D. Taylor-Robinson

**Affiliations:** 1 Excellence & Friends Management Care Centre (EFMC), P. O. Box 200, PSIN Dutse, Abuja, Nigeria; 2 APIN Public Health Initiatives, Ltd., Abuja, Nigeria; 3 Hepatology Unit, Imperial College London St Mary’s Hospital Campus, 10th Floor, QEQM Building, South Wharf Road, London, W2 1NY United Kingdom

**Keywords:** Healthcare workers, Physicians, Industrial actions, Strike, Nigeria

## Abstract

**Background:**

The Nigerian health system has been plagued with numerous healthcare worker strikes (industrial action) at all levels. The purpose of this study is to document physicians’ views on healthcare worker-initiated strike action in Nigeria and represent a follow-on to a previous study where poor leadership and management were cited as the most common cause of strike action by healthcare workers.

**Methods:**

A cross-sectional, descriptive study was executed between April and June 2017. We used a self-administered pre-tested structured questionnaire with open-ended questions to allow for better expression of participants’ views. Participants were drawn mainly from the recently concluded West African College of Physicians (WACP)/Royal College of Physicians (RCP) Millennium Development Goal 6 Partnership for African Clinical Training (M-PACT) course. They represented the six geopolitical zones of Nigeria. Data were analysed using SPSS v 23. Simple frequencies were performed, and relevant tables/charts were developed.

**Results:**

A total of 58 physicians (out of 131 participants reached) responded to the study, giving a response rate of 44.3%. 62.1% were males, 67.9% were between the ages of 30 and 39 years, and over 60% of respondents graduated prior to 2010. Poor staff welfare was cited by 16.7% as the commonest cause of strikes in the healthcare system. Other causes cited were salary issues (13.9%), leadership and management (13.9%), poor hospital infrastructure (11.1%), poor guidelines and services (11.1% each) and inter-professional disputes (5.6%). The negative consequences of strikes, the groups who benefit from them and solutions to the strikes were enumerated, including training physicians in leadership skills by 98.2% of respondents.

**Conclusion:**

Poor staff welfare, salary and leadership/management and governmental inability to implement agreements were the common causes of healthcare worker strikes in this study. These strikes resulted in disruption to service delivery and training programmes, increased morbidity and mortality of patients and loss of confidence in the hospitals and the healthcare professions. The participants recommended that the Federal Government respects agreements made with the management of healthcare institutions, implements the National Health Act and ensures that only leaders and managers who are formally trained are appointed to healthcare management positions.

## Background

The healthcare provision in Nigeria has suffered greatly from numerous healthcare worker strikes (industrial action) over the years. These have resulted in multiple avoidable mortalities and morbidities in Nigeria, further destroying the already poor health outcomes in the country. As documented in an earlier study, strikes have remained common occurrences in Nigeria as there are local, state, regional and sometimes national industrial action on a regular basis [1]. A recent review shows that between April 2016 and April 2017, there were at least 17 different strike actions in Nigeria involving public workers [2–6].

**Table:** 

S/No	Description	Date
1	All Imo State Doctors	February 2016
2	Imo State University	March 2016
3	Kogi State University	April 2016
4	University of Lagos	April 2016
5	Adekunle Ajasin University, Akungba-Akoko (AAUA)	April 2016
6	University of Ibadan	April 2016
7	Obafemi Awolowo University	June 2016
8	Federal Medical Centre, Ondo	Mid 2016
9	Ladoke Akintola University	June 2016
10	Federal University of Agriculture Abeokuta	August 2016
11	Ebonyi State University	October 2016
12	Ambrose Ali University	October 2016
13	Nigeria Union of Petroleum and Natural Gas and PENGASSAN	January 2017
14	National Orthopaedic Hospital, Igbobi, Lagos (NOHIL)	March 2017
15	Nigerian Union of Allied Health Professionals (dental therapists, medical physicists, health information officers, clinical psychologists and medical social workers)	March 2017
16	Federal Medical Centre, Yenogoa	May 2017
17	Benue State University Teaching Hospital Joint Health Sector Union (JOHESU) chapter	May 2017

**Fig. 1:**
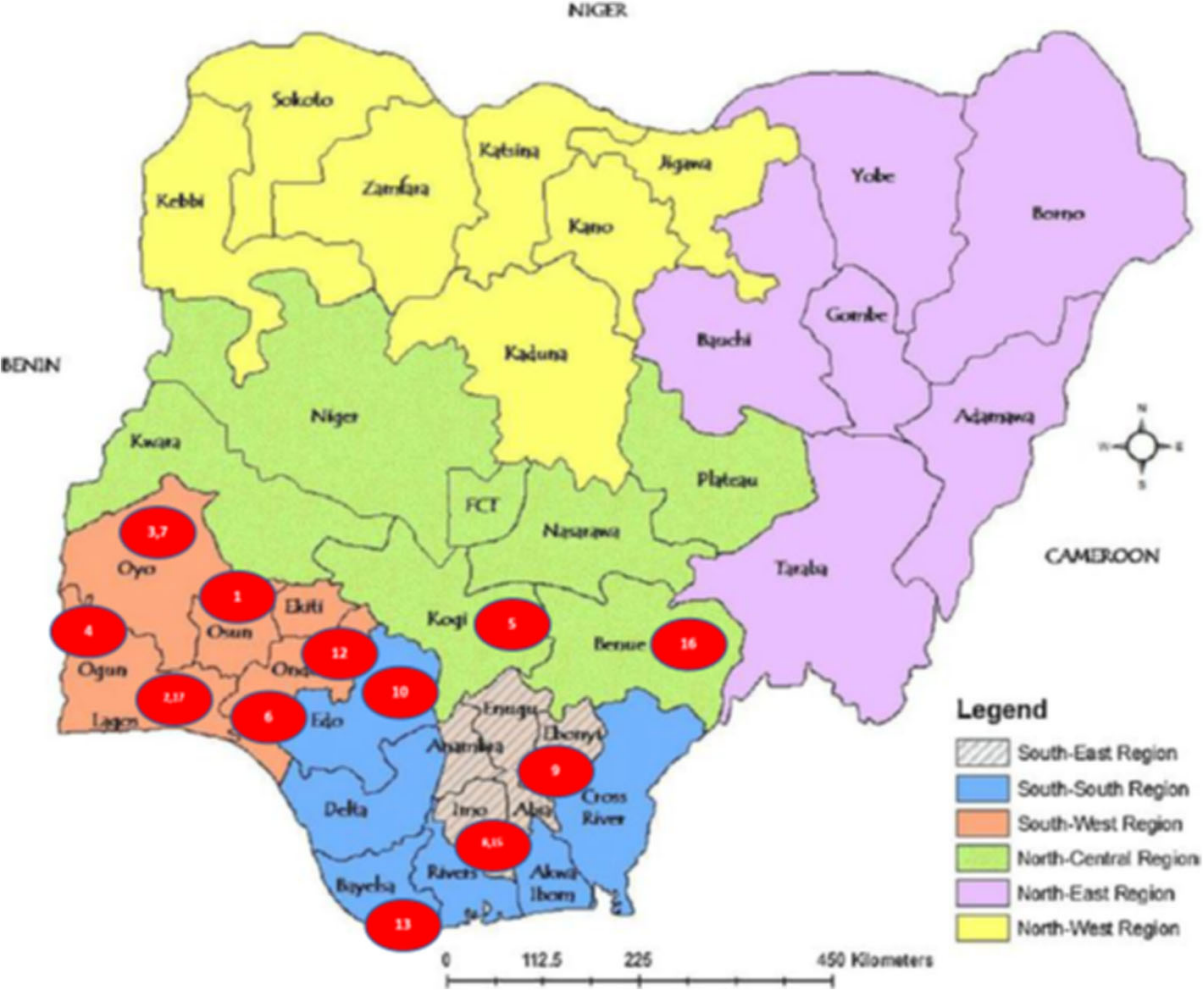
Map showing locations of strike actions in Nigeria

Of the 17 documented and reported strike actions, six were primarily within the healthcare establishment. Also, apart from two national industrial actions—Nigeria Union of Petroleum and Natural Gas Workers (NUPENG) and Nigerian Union of Allied Health Professionals strikes—(affecting all states and regions of the federation), the rest occurred in the southern region of Nigeria as shown in Fig. 1.

**Table:** 

1- Obafemi Awolowo University	9- Ebonyi State University
2- University of Lagos	10- Ambrose Ali University
3- Ladoke Akintola University	15- Imo State
4- Federal University of Agriculture, Abeokuta	16- Benue State University Teaching Hospital
5- Kogi State University	17- National Orthopaedic Hospital, Igbobi, Lagos
6- Adekunle Ajasin University, Akungba-Akoko	
7- University of Ibadan	
8- Imo State University	

In 2014, we reported on a cross-sectional study among healthcare workers in Nigeria. We studied a total of 150 healthcare workers, of which less than half (43.6%) supported industrial action [1]. We reported that healthcare leadership and management were cited as the most common (92%), as well as the most important (43.3%), cause of healthcare worker industrial action in Nigeria [1]. Other cited causes were a demand for higher salaries and wages (82%), infrastructural issues (63.3%) and interpersonal issues (61.3%). However, only 2.0% of respondents rated current healthcare management as excellent, while 24.0% rated it as very good.

Since the study involved different healthcare professionals, we decided to focus further investigation on physicians, who also were most implicated in the leadership challenge. Also, since the study concentrated on healthcare workers in Abuja, the Federal Capital Territory, we planned to involve all the regions of Nigeria in an expanded study. The aim of the study was to identify and document the frequency of physician-initiated strike actions in healthcare facilities and the views of physicians regarding these strike actions in terms of cause, effects and outcomes of the strikes.

## Methods

We used a cross-sectional study design. Study populations were selected from physicians who attended the recently concluded West African College of Physicians (WACP)/Royal College of Physicians (RCP) Millennium Development Goal 6 Partnership for African Clinical Training (M-PACT) course, funded by the EcoBank Foundation in Nigeria. Such sampling ensured inclusion of participants from all the geopolitical regions of Nigeria. Participants were informed of the nature of the study and participation was by choice as non-participation was not associated with any negative consequences.

We used a self-administered, pre-tested for accuracy, analysability, acceptability and ambiguity, and properly tailored questionnaire. The pre-test was done among physicians who were not part of the selected study population, and issues of ambiguity noticed in the questionnaire were corrected before the final study was carried out. The questionnaire had 23 different questions and the majority were open-ended to allow for proper participants’ expressions of their views and to prevent biased responses from single option or closed questions. Approval for the study was obtained from the Board, Excellence and Friends Management Care Centre (EFMC) and respective partners in the United Kingdom (Royal College of Physicians of London).

Questionnaires were distributed through participants’ emails, and each individual was given 14 days to respond to the questionnaires. No reminder email was sent, as this was also a test of participants’ willingness to be part of a changed healthcare system.

Completed questionnaires were collected and retrieved online, screened for accuracy and completeness, and analysed using SPSS v 23 (SPSS Inc., Chicago, Illinois, USA). Simple frequencies were performed, and relevant tables/charts were developed. Sex, age, educational qualification, job status and the number of years in service were used in the analysis.

## Results

A total of 58 physicians (out of 131 participants in the Nigerian courses, drawn from the first 2 years of the programme) responded to the study, giving a response rate of 44.3%.

### Demographic characteristics of respondents

Males constituted 62.1% of the respondents, and the majority were between 30 and 49 years of age (Table 1). Over 60% graduated before 2010, and of the 57 who responded to the question on their qualifications, 42.1% had additional qualifications and over 33.4% had a membership or fellowship qualifications of the West African College of Physicians. The majority of the participants were from the South South (14, 24.1%) and South West (18. 31.0%) geopolitical regions of Nigeria. The least represented region was the North East, where there has been an ongoing insurgency since 2010. Two non-Nigerians who attended the Nigerian courses also responded to the survey (Table 1).

**Table 1:** Demographic characteristics of respondents

	Frequency	Percent
Age of respondents (years)		
< 30	6	10.7
30–39	38	67.9
40–49	11	19.6
50–59	1	1.8
Total	56	100
Missing system	2	3.4
Year of graduation of respondents		
< 2000	2	3.6
2000–2004	7	12.5
2005–2009	35	62.5
2010–2014	10	17.9
2015 +	2	3.6
Total	56	100
Missing system	2	3.4
Academic qualification of respondents		
MBBS/BDS	57	100
MPH or MSc	7	12.3
MWACP	16	28.1
FWACP	3	5.3
Total	57	98.3
Missing system	1	1.7
Total	58	100
Geopolitical zones of participants		
North Central	8	14.3
North East	1	1.8
North West	4	7.1
South East	9	16.1
South South	14	25.0
South West	18	32.1
Non-Nigerian	2	3.6
Total	56	100
Missing system	2	3.4
Department/faculty of respondents		
Internal Medicine	15	27.3
Community Health	16	29.1
Family Medicine	7	12.7
General Practitioner	12	21.8
Paediatrics	3	5.5
Others	2	3.6
Total	55	100.0
Missing system	3	5.2
Position/level in training		
Medical Officer of Health/Registrar	14	24.6
Senior Registrar	39	68.4
Consultant/Fellow	3	5.3
Others	1	1.7
Total	57	100
Missing system	1	24.6

Internal medicine (12, 27.3%) and community health (16, 29.1%) constituted the largest portion of the respondents.

### Frequency, nature and consequences of industrial action in healthcare establishments

**Fig. 2:**
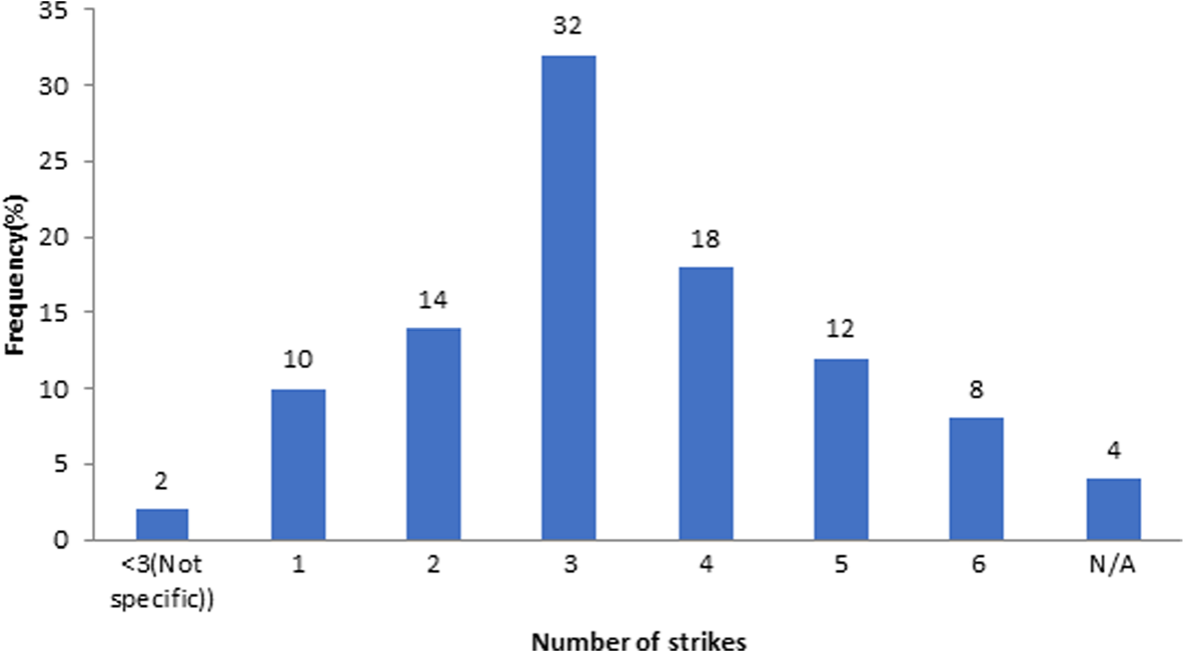
Number of healthcare workers’ strikes/industrial actions that took place in healthcare institution across Nigeria in 2016

In 2016, there were between 0 and 6 different strike actions in each Nigerian healthcare institution with three strikes (3) as the most frequent number of industrial action taken (Fig. 2).

Over the past 3 years, participants reported that there has been between 1 and 15 different strike actions in the healthcare systems, with 88% claiming that there has been over six different strike actions and 16% over 11 strike actions. Of these, 96.0% believed that at least one of the strikes was championed by doctors, with 19.2% believing that physicians were responsible for three or more strike actions in the year (Table 2).

**Table 2:** Physician and other healthcare workers’ mediated strike actions in Nigeria in 2016

	Frequency	Percent	V. Percent
Physicians mediated healthcare worker strikes in Nigeria			
0	2	3.4	4.3
1	14	24.1	29.8
2	22	37.9	46.8
3	5	8.6	10.6
4	2	3.4	4.3
5	2	3.4	4.3
Total	47	81	100
Missing system	11	19	
Other healthcare workers mediated strike actions in 2016			
0	2	4.45	4.45
1	20	44.45	44.45
2	17	37.8	37.8
3	5	11.1	11.1
4	1	2.2	2.2
Total	45	100	100
Missing system	13	22.4	

The commonest cause of strike in the healthcare care cited by 16.7% of the participants was staff welfare (Table 3). Compensation and salary issues, government, and leadership and management were cited by 13.9% as the next common cause of industrial action in healthcare in Nigeria. This was followed by poor infrastructure (11.1%), poor guidelines and services (11.1% each) and inter-professional disputes (5.6%).

**Table 3:** Common causes of healthcare worker strikes in Nigeria

Description	Frequency	%	Type of comments made
Government	5	13.9	Failure of hospital management to uphold Federal Government (FG) agreements about salaries and allowances; Breach of trust by the government and government insincerity; Unfulfilled agreements reached with the Government on wages, training, equipping the hospital; Insincerity of FG and Chief Medical Directors (CMDs) in keeping to agreements and manipulation of union heads by politicians; Change of Government
Infrastructures	4	11.1	Poor hospital utility facilities (No water in wards, Poor/No doctors’ call room, etc.); Poor working environment; Security of staff; and Poor infrastructure in the hospitals
Leadership and management	5	13.9	At the core of it is welfare issues like low staff motivation; Poor communication between hospital leadership/administration and healthcare workers; Hospital leadership quick to respond to circulars from the Federal Ministry of Health (FMOH) that are anti-workers, but slow to respond to circulars that favour workers; Hospital leadership disconnected from the true situation of things in the hospital and unmindful of the experiences of patients; Leadership tussle
Welfare	6	16.7	For improvement in workers’ welfare; Delayed promotion; Joint Health Sector Union’s (JOHESU) demand to be appointed consultants; Failure of the management to sponsor residents’ exams and updates; Denial of basic entitlement such as salary and training sponsorship
Compensation and salary	5	13.9	Poor workers’ compensation; Inconsistent salaries; Delayed salary payment; Pay check is delayed and very small to meet up with present day reality; Remuneration skipping/relativity
Inter-professional disputes	2	5.6	Inter-professional disputes, unnecessary disharmony amongst healthcare workers
Poor guidelines and services	4	11.1	Poor guidelines for progression for different cadre of healthcare workers; Agitation for improved service delivery; Upgrading the standard of health care delivery and assault on a health worker; Lack of residency training policy
Others	5	13.9	Lack of facilities, residency templates for residents; In solidarity with unjustly dismissed colleagues in order that they be reinstated; For regularization of entry and other grade levels; Greed; Protest against sack of residents who have overstayed

Healthcare worker strikes have various consequences. We classified these according to themes into seven main groups (Service Delivery impacts, Morbidity and Mortality impacts, Institutional impacts, Professional impacts; Healthcare outcomes impacts, Governmental impacts, and Training impacts) as shown in Table 4.

**Table 4:** Common consequences of healthcare worker strikes in Nigeria

Description	Comments on strike consequences
Service delivery impacts	Skeletal services; Patients are unable to access healthcare services; Poor patient care; Patients suffer as patients that need attention are denied specialist attention; Beneficial departmental activities are stalled/cancelled; Disruption in service rendition
Morbidity and mortality impacts	Loss of lives; Increased morbidity and its complications, increase in maternal and child mortality, overall mortality and morbidity: Patients who cannot afford private facility bills may end up dying
Institutional impacts	Reduced revenue generation by the hospitals; Loss of confidence in the system; Industrial actions weaken the health system and constantly dragging the system backwards; negatively affects the productivity of a particular institution; Increased distrust of the public health system by the general public, dissatisfaction of clients/patients; Conflicts between employees and management: Reduced internally generated revenues; Disruption in key health services including immunization and Prevention of Mother-to-child Transmission of HIV (PMTCT) services; Low productivity
Professional impacts	Loss of dignity and respect for the profession; Poor public perception of medical personnel; Unhappy doctor; Reduced efficiency of human and material resources; Unhealthy inter- and intra-health workers relationships
Healthcare outcomes impacts	Distortions of patient care outcome parameters (both clinical and patient satisfaction)
Governmental impacts	Government agencies responsible for reason for strike actions suddenly wake up to respond to the agitated needs; Medical tourism and capital flight
Training impacts	Residents, especially in clinical departments, do not get to do enough procedures to meet the requirement of the various colleges in good time; Poor performances in postgraduate examinations with huge financial losses; Delayed clinical posting of medical students leading to overstay in the university; Disruption of research activities

Although healthcare worker strikes have several negative consequences, they are also believed to be beneficial to a number of people including healthcare workers (21.8%), private practitioners (21.8%), union leaders (5.5%) and sometimes the Government. However, the patients have suffered the most, according to 83.9% of the respondents (Table 5).

**Table 5:** Groups and individuals that benefit from or are impacted by healthcare worker strikes

Description	Frequency	Percent
Groups and individuals that benefits from healthcare worker strikes		
No one	26	47.3
Healthcare workers	12	21.8
Private hospitals/practitioners	12	21.8
Union leaders	3	5.5
Not sure	2	3.6
Government	3	5.5
Patient	1	1.8
Consultants	1	1.8
Groups and individuals negatively impacted by healthcare worker strikes		
Patients	47	83.9
Patients and their relatives	3	5.4
General public	6	10.9
Residents doctors	6	10.9
Poor socio-economic class	3	5.4
Government	2	3.6

### Proposed solutions to healthcare worker strikes in Nigeria

On what could be done to stop or minimize the number of healthcare worker strikes in Nigeria in any given year, the respondents were of the view that the Government should respect agreements and implement the Nigerian National Health Act; hospital management and leaders should run an all-inclusive government with better communication strategies; healthcare workers should be actively involved and represented in decision making and management; salaries and wages should be improved and paid on time; and infrastructure and training systems should be improved upon. See Table 6 for sample unedited comments.

**Table 6:** Suggested solutions to national healthcare worker strikes in Nigeria

Theme	Samples of comments on solutions to healthcare worker strikes
Government respect for agreements and health acts	Government should implement the signed agreements, not just put them to paper; Fulfilment of agreement by Government to all health workers; Improvement in health service delivery including implementation of 2014 National Health Act. More sincerity on the part of Government
Better leadership and management systems	Hospital management should communicate better with residents (not by issuing threats); The hospitals should operate at an internationally acceptable standard for the kind/level of facility it is; The hospital administration/leadership should involve the healthcare workers in decision making, leadership should be inclusive; To work on the leadership and administration of the health system; Sincerity on the part of hospital management
Improved inter-professional relationships	Mutual respect among all healthcare cadres
Timely and better salaries and compensations	Consistent and regular payment of salaries; Regularization of salaries to remove inconsistency; The remuneration of the health worker should be given topmost priority by the government and if they are unwilling, they can privatize hospitals for better management
Infrastructural upgrade	Improved infrastructure in hospitals; Improved working conditions—renovations of the call rooms, Make working environment conducive
Improve staff welfare	Increase welfare of health workers
Better training management systems	There should be a clear template for the residency training programme which is universally applicable in all facilities in the country, and this should be followed strictly, including staff strength, work duration(s), remunerations, and taxation

Also, 98.2% of the respondents were of the view that every physician should be trained in leadership skills. To achieve this, 87.3% were of the opinion that training in leadership should occur at both undergraduate and graduate/fellowship levels. The rest 5.5% and 7.3% wanted the training at undergraduate and postgraduate levels, respectively. To achieve this, they identified major deficiencies in the current medical curriculum which they said was outdated, was too didactic and does not allow for lecturer-student relationship nor stimulate accountability in lectures, and has no training on leadership and entrepreneurship. Other obstacles identified in the current training system are documented in Table 7.

**Table 7:** Major deficiencies in the current curriculum for medical students

Theme	Comments on training deficiencies
Faculty and lecturers	Poor quality and mindset of the teachers (a lot of the teachers were seen as being not ethical in their leadership conduct and this has had a negative effect on the students, i.e. bullying behaviour during teaching sessions from consultants and registrars.); Lack of/Poor mentorship; Negligence of meritocracy; Poor staffing; Lack of commitment of consultants to residency training; Consultants were seen as being too busy to impact sound training for residents; Poor mentorship and commitment to residency training; Poor supervision by the trainers
Funding	There is also the challenge of poor funding, obsolete teaching methods and facilities; Inadequate funding; Poor health system funding.
Infrastructure	Overcrowded classrooms, poor amenities, too many distractions for medical students; Lack of essential equipment for skill acquisition; Lack of/Poor medical infrastructure; Lack of adequate facilities even in accredited institutions; Lack of equipment
Research and development	Lack of sponsorship for research in tertiary institutions; The major deficiencies are lack of investigative materials.
Curriculum	Lack of leadership and administrative training in the medical training; Deficiencies in curriculum, deficiencies in training guidelines, deficiencies in training institutions, on-appraisal of what is existing already and creating a benchmark for operation; Lack of harmonization of training curriculum; Non-inclusion of leadership/management training, policy-making and research with hands-on experience even at the postgraduate level especially as doctors are expected to lead the health team
Career path	Lack of job security during and after the programme; Spaces for new fellows seem to be shrinking; The exams were considered to be biased; More emphasis on passing exams than on skill acquisition
Strikes	Frequency of strikes; Incessant interruptions during the period of residency training; Interruption/ prolongation of residency training programme

A respondent said, “Truly, medical training in Nigeria does not equip the doctor to lead beyond the clinical ward round sessions. The mentality of statutory leadership by doctors is currently being threatened because of the significant failure of the medical doctors who have and are still ‘ruling’ the sector. They have erroneously transferred the clinical model of patient care leadership to hospital management and the result is what we have today. Deliberate leadership and management training must be incorporated into the curriculum.”

**Fig. 3:**
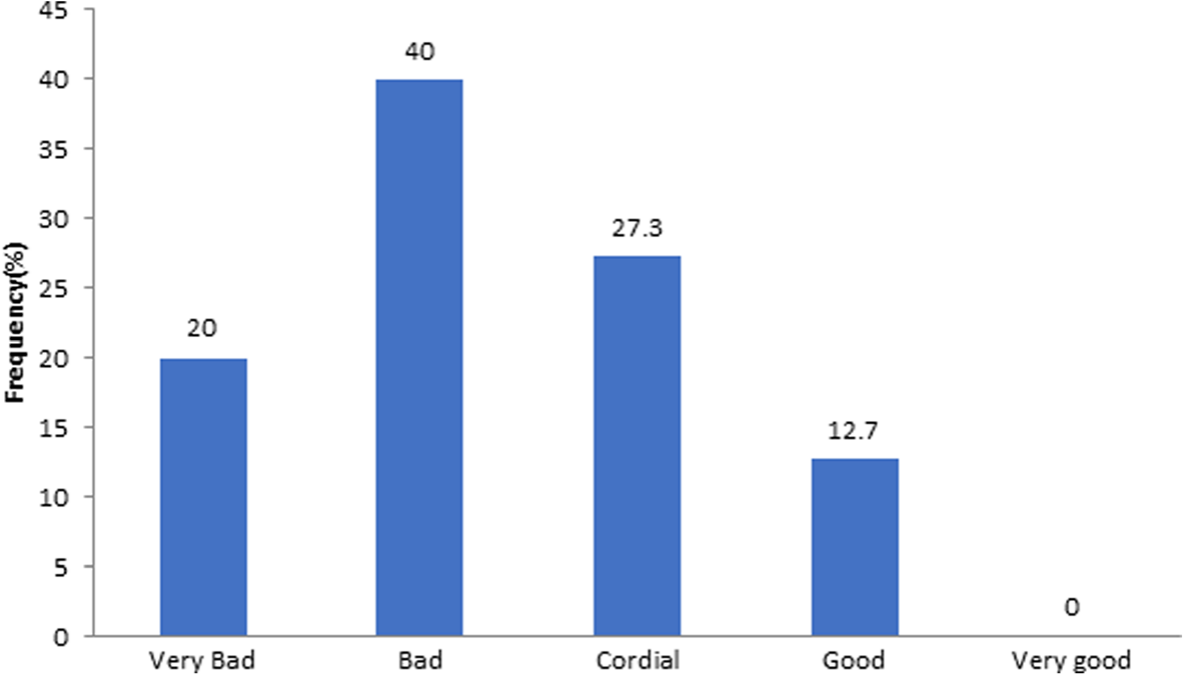
Current relationship between doctors and hospital management

This was critical because for 60% of the respondents, the relationship between doctors and the employing institution was considered to be very bad (20%) and bad (40%), with only 12.7% saying it was good. No one said it was very good or excellent (Fig. 3).

## Discussion

Although the response rate was poor, the findings of this study are very instructive. The study catalogues the thinking of medical doctors who have had the privilege to lead healthcare systems in Nigeria for a very long time, and who are still in leadership roles.

While over 82% of the respondents graduated from medical school on or before 2009, none had a definable leadership and management certificate/qualification. This is more informative as their subsequent comments showed that there had been little or no leadership and management education in their undergraduate and postgraduate training.

The very low response rate from North East of Nigeria could be a reflection of the ongoing insurgency in the area, which has caused many healthcare workers to flee the region, delaying patient access to quality healthcare as and when needed, and led to increased cost of healthcare, and morbidity and mortality in the area.

The higher rate of response from Medical Officers of Health/registrars and senior registrars in internal medicine and community health is probably a result of their higher representation in the WACP/RCP courses in Nigeria (from which the study participants were drawn), as they formed a major percentage of trained physicians over the period of study. The views captured in this study may also reflect their understanding of the system and their level of training. For future studies, it would be instructive to study more senior doctors including consultants and fellows to compare their views with those of the junior officers highlighted in this study.

This study shows that healthcare workers’ strikes were common across the nation—but majority occurred in the southern regions. There were no public healthcare workers in the country who had not embarked on one or more episodes of industrial action during the period under review with its attendant consequences. This, we believe, is both disruptive and detrimental to the healthcare system in Nigeria. Furthermore, the majority of the strike actions were initiated by physicians, who also lead the healthcare facilities, thus, undermining indirectly, what they are mandated to lead and maintain.

The key causes of healthcare workers’ industrial actions mentioned by the respondents included Government’s inability to implement agreements, infrastructural challenges, poor leadership and management with inadequate communication strategies, poor staff welfare, inadequate and delayed salaries, inter-professional disputes and poor adherence to practice guidelines and policies. These are similar to findings from our earlier study [1]. Although there was not a marked difference in the number of respondents who cited leadership and management from the rest, if staff welfare challenges such as delayed promotion, failure of the management to sponsor residents’ exams and updates, denial of basic entitlement such as salary, and training sponsorship were classified as leadership and management problems, leadership and management can be seen to be the most common cause of healthcare workers’ industrial action as documented before [1].

Healthcare workers’ strikes have several devastating consequences on the healthcare system including disruption in service delivery, increased morbidity and mortality of patients, reduced revenue generation for hospitals, loss of confidence in hospitals and the healthcare profession, poor patient care outcome parameters, and disruption of training programmes with longer stay of residences within the healthcare system. Apart from a possible improvement in governmental engagement and conversation with healthcare workers, all outcomes of strikes are seen to be negative and highly disruptive to the healthcare system. Other respondents’ documented benefits to healthcare workers, private practitioners and government may be seen to be detriment of the patient who suffers most from all strike actions, as noted by 83.9% of the respondents.

A study was carried out during a 20-day strike in a hospital in South Africa, and it provided evidence of significantly lowered quality of service delivery with an overall reduction in the number of admissions and surgeries performed in the hospital when compared to a non-striking situation as hospital mortality, when correlated with number of hospital admissions, increased threefold for the hospital as a whole while mortality doubled in the surgical department during strike actions [7]. However, a review of the impact of healthcare strikes in other parts of the world, especially in developed countries, has shown that healthcare workers’ strikes may not significantly affect the health of patients, especially if there is the availability of emergency services or affordable private fee-for-service care [8]. By contrast, this is not the case in Nigeria as there have never been contingency plans for the welfare of the patients before and during these strikes.

Studies conducted in hospitals in Sweden, Israel and the United States of America during periods of strike action showed an increase in patient volume at emergency units for emergency care, diagnosis and treatment [8–10]. Mortality rates either remained constant or decreased, due to a reduction in the number of elective surgeries performed [8, 11]. Although there were no deaths from elective surgeries because they were cancelled, it was observed that there was an increased number of deaths, which occurred during transfers between emergency rooms [11]. Studies have also shown that patients from lower socio-economic strata of the society are less able to cope with healthcare workers’ strikes probably because they are unable to afford alternative or private healthcare services [8, 11]. These underline the need to eliminate healthcare worker strikes as they result in avoidable deaths and morbidities.

In this study, several mitigating steps were suggested and these include that the Government (at all levels) should respect agreements and implement the Nigerian National Health Act, as also recommended in a case study by Adeloye et al. [12]. The National Health Act intends to positively impact universal health coverage, access to and cost of healthcare, funding and insurance facilities, practice by healthcare providers, quality and standards, patient care and health outcomes [12]. Respondents in our study also believe that there should be provision of better leadership and management systems for the healthcare industry; workers and Government should partner to improve inter-professional relationships, while salaries and financial remuneration should be timely and appropriate. Other welfare matters should be investigated in a timely fashion and improved upon, workplaces should be made more appropriate with infrastructural upgrade and provision of relevant equipment, and training programmes should be standardized with appropriate clinical and practice guidelines, policies and standards of practice developed.

To minimize strikes, the respondents suggested that hospital management should communicate better with residents and that hospitals should operate at an internationally accepted standard for the kind/level of facility it is. This includes involving healthcare workers in decision making; leadership should be inclusive; mutual respect among all healthcare cadres; consistent and regular payment of salaries and regularization of salaries to remove inconsistency; improved infrastructure in hospitals with better working conditions; and establishment of a clear template for the residency training programme which is universally applicable in all facilities in the country.

The Joint Learning Initiative on Human Resources for Health and Development, Report, in response to the demotivation of healthcare workers which moves them to strike action, states that “a key action is a significant upward revision of the total compensation package to a level that reflects the value placed on the work they do, is likely to discourage staff from wanting to leave public sector services” [13]. Moreover, to improve the leadership and management standards of the industry which will drastically reduce the number of healthcare workers who strike, over 98% of the respondents were of the view that every physician should be trained in leadership skills at both undergraduate and graduate/fellowship levels. This will require modification of the current medical curriculum, improvement in lecturer-student relationships and improvement in the level of accountability of lecturers. Also, as managers of finances and materials resources, physicians should be trained in financial management and entrepreneurship.

We agree with Adeloye et al. that the Nigerian health system requires a solid administrative policy foundation that will allow for alignment and coordination of partnerships among various stakeholders in the health workforce [12]. We believe that to develop competency in public health leadership, public health leaders require competency-based instruction to increase their ability to address complex and changing demands for critical services [14]. With a spirit of cooperation, it should be possible to minimize or completely eliminate healthcare workers’ strikes in Nigeria.

### Limitations

Online distribution of questionnaire for a study has its various limitations which include poor response rates. Although this was noticed in this study, a response rate above 40% is significant and deductions made are statistically viable. We could not study the characteristics of the non-responders to determine if they were different from the responders. Also, we focused on physicians who just attended a course. This may have influenced their views and understanding of physician leadership roles.

Convenience sample was used for this study. In addition, the study population is limited to only physicians who attended the course. Therefore, the results of this study may not be generalized to all doctors in Nigeria because surgeons and other specialist were not included in the study.

As the study was small and limited in nature, it was supposed to be approved by the Board of Excellence and Friends Management Care Centre (EFMC), but as two of the authors are also Board members, this may create “a potential conflict of interest”. So, to avoid this, the EFMC Board members were not part of those that approved the work. The national ethical committee will approve subsequent works in this area.

## Conclusions

This cross-sectional study has again highlighted the common causes and consequences of healthcare worker strike actions in Nigeria. To minimize these, the Federal Government must respect all agreements made with the management of healthcare institutions, implement the National Health Act, ensure that only appropriately trained leaders and managers are given the responsibility of managing the hospitals in the country, work to ensure that salaries and financial remuneration are fair and paid on time, improve the work environment by providing necessary work tools and equipment and improve the welfare of staff, especially those working in difficult environments. The Government should also standardize training programmes in all institutions.

Although the Government has a significant role to play, physicians who lead must lead well, ensuring that they acquire the right set of skills and competencies in leadership through formal and tailored health policy, leadership and management training, and manage the healthcare system dispassionately with fairness. Nigerian medical professionals must all work together to minimize the disruption to healthcare service delivery, with the attendant morbidity and mortality. Improvement of institutional, professional and healthcare outcomes can only be brought about in a spirit of collaboration. This will help rebuild the lost confidence of the people in the healthcare system and improve community health.

Future studies should also investigate other ethical aspects of the practice of medicine in this environment and how the impact of training in medical ethics or ethics of the healthcare professions in the current medical curricula in Nigeria, or lack thereof, might influence the frequent incidence strikes in Nigeria.

## Abbreviations

FG: Federal Government
JOHESU: The Joint Health Sector Union
M-PACT: Millennium Development Goal 6 Partnership for Clinical Training
RCP: Royal College of Physicians
WACP: West African College of Physicians
